# Cereal processing at Early Neolithic Göbekli Tepe, southeastern Turkey

**DOI:** 10.1371/journal.pone.0215214

**Published:** 2019-05-01

**Authors:** Laura Dietrich, Julia Meister, Oliver Dietrich, Jens Notroff, Janika Kiep, Julia Heeb, André Beuger, Brigitta Schütt

**Affiliations:** 1 German Archaeological Institute, Orient Department, Berlin, Germany; 2 University of Würzburg, Institute of Geography and Geology, Würzburg, Germany; 3 Freie Universität Berlin, Institute of Geographical Sciences, Berlin, Germany; 4 Museum Düppel, Berlin, Germany; University at Buffalo - The State University of New York, UNITED STATES

## Abstract

We analyze the processing of cereals and its role at Early Neolithic Göbekli Tepe, southeastern Anatolia (10th / 9th millennium BC), a site that has aroused much debate in archaeological discourse. To date, only zooarchaeological evidence has been discussed in regard to the subsistence of its builders. Göbekli Tepe consists of monumental round to oval buildings, erected in an earlier phase, and smaller rectangular buildings, built around them in a partially contemporaneous and later phase. The monumental buildings are best known as they were in the focus of research. They are around 20 m in diameter and have stone pillars that are up to 5.5 m high and often richly decorated. The rectangular buildings are smaller and–in some cases–have up to 2 m high, mostly undecorated, pillars. Especially striking is the number of tools related to food processing, including grinding slabs/bowls, handstones, pestles, and mortars, which have not been studied before. We analyzed more than 7000 artifacts for the present contribution. The high frequency of artifacts is unusual for contemporary sites in the region. Using an integrated approach of formal, experimental, and macro- / microscopical use-wear analyses we show that Neolithic people at Göbekli Tepe have produced standardized and efficient grinding tools, most of which have been used for the processing of cereals. Additional phytolith analysis confirms the massive presence of cereals at the site, filling the gap left by the weakly preserved charred macro-rests. The organization of work and food supply has always been a central question of research into Göbekli Tepe, as the construction and maintenance of the monumental architecture would have necessitated a considerable work force. Contextual analyses of the distribution of the elements of the grinding kit on site highlight a clear link between plant food preparation and the rectangular buildings and indicate clear delimitations of working areas for food production on the terraces the structures lie on, surrounding the circular buildings. There is evidence for extensive plant food processing and archaeozoological data hint at large-scale hunting of gazelle between midsummer and autumn. As no large storage facilities have been identified, we argue for a production of food for immediate use and interpret these seasonal peaks in activity at the site as evidence for the organization of large work feasts.

## Introduction

Cereal food is one of the main components of the modern human diet. Its integration into the subsistence strategy during the late Epipalaeolithic (c. 12500–9600 cal BC) and Pre-Pottery Neolithic (PPN, c. 9600–7000 cal BC) has been recognized as a very long and complex process involving the selection and utilization of plants, strategies of exploitation of plants and land, the development of cultivation, and ways of processing, storing and consuming plants [1–12]. The establishment of agricultural economies at the end of the later part of the Pre-Pottery Neolithic (PPNB, c. 8800–7000 cal BC), comprising the deliberate, large-scale cultivation of domesticated cereals and other domesticated plants [1, 13–16], was predated by a longer period of experimentation and technological modifications that led to the development of a specialized tool kit for plant food processing [17–24]. Typical implements for cereal processing are pounding and grinding tools used in pairs, comprising a static low implement (mortar, grinding slab or grinding bowl) and an active upper tool that is moved across its surface (pestle or handstone) [25]. The different processes for fragmenting cereals include de-hulling, pearling, polishing or grinding to fine flour and are also ethnographically attested [26]. The aim of all these techniques is to enhance the digestibility of cereals, lower their cooking time and raise their dietary energy [27]. Early direct evidence for the processing of cereals to fine flour through grinding was found in the Early Epipaleolithic site of Ohalo II, dated to c. 21000 cal BC [20, 27, 28] and the Early to Late Natufian site of Shubayqa, dated to 12500–9600 cal BC [29]. However, the regular processing of wild cereals through grinding seems to have been established first in the Late Natufian, as suggested by macrobotanical evidence [11, 30] as well as by morphological changes in grinding stones combined with use-wear analyses [17]. The increase of grinding stones with typical use-wear in Late Natufian contexts [17, 18] and the reduction of forms of bed-rock mortars at the end of the Natufian and during the PPNA (c. 8800–7000 cal BC) [31] can be interpreted as indicating increased processing of cereals as food sources and the establishment of grinding as a more effective processing technique. New analyses seem to confirm the important role these features played in the processing of cereals and the production of beer in the Late Natufian [32, 33]. Flat, large grinding stones and handstones became a supra-regional standard during the subsequent Levantine PPN, constituting an integral part of the architecture [21, 22, 30]. This development seems to coincide with the general trend of increasing use and production of cereals [1–12, 34]. However, there was significant regional variability in the establishment of cereals as one of the main food sources [2, 9].

Both mortars / pestles and handstones / grinding slabs / bowls can be used for the processing of cereals like a variety of ethnographic examples show [26]. Many factors, like the available raw material, customs or technological knowledge can theoretically influence changes in tool morphologies [18]. The functions of grinding stones therefore have to be established using an integrated program of contextual, formal and use-wear analyses.

Recent investigations have highlighted the area between the upper reaches of Euphrates and Tigris as *one* region where the transition to food-producing subsistence took place early during the Epipalaeolithic and the Pre-Pottery Neolithic. The distribution areas of the wild forms of einkorn, emmer wheat, barley and other ‘Neolithic founder crops’ overlap here and DNA fingerprinting has pinpointed the transition of two wild wheat variants to domesticated crops to this part of the Fertile Crescent [35–39]. Systematic early plant use has been found at a variety of sites, like Cafer Höyük [40], Çayönü [41], Hallan Çemi [42], Jerf el Ahmar [30], and Körtik Tepe [43, 44]. Some of these sites have produced large quantities, in some cases several hundreds, of items for plant food processing (handstones, large grinding bowls and slabs as well as mortars and pestles) [30, 45].

Typical early PPN residential structures are small, round to oval and semi-subterranean huts, which are later, during the PPNB, replaced by planned settlements with large rectangular buildings [46–48]. Already at the start of the PPN, ‘special buildings’, often monumental in nature and with a common iconography, appear in Upper Mesopotamia at sites like Çayönü, Dja´de al Mughara, Göbekli Tepe, Gusir Höyük, Hallan Çemi, Jerf el Ahmar, Mureybet, and Nevalı Çori [47–70]. These ‘special’ buildings have been interpreted as being related to ritual [52, 53, 56, 57, 64, 68], as places that served as ‘external memorial storage’ for the societies constructing them [71], or as ‘communal buildings’ for a variety of tasks [59, 66]. A connection of (some) ‘special buildings’ with cereal use has been stressed especially for one site so far. At PPNA Jerf el Ahmar, subterranean circular ‘communal’ buildings were surrounded by residential architecture [66]. One of them, building EA 30, was a round semi-subterranean construction divided into cells [30]. As these cells contained higher than average concentrations of cereals (mostly barley), the building was interpreted as a communal silo [30]. It was surrounded by rectangular limestone buildings, which in some cases showed installations of several querns in a row.

The by far largest assembly of ‘special buildings’ so far known is that of Göbekli Tepe in southeastern Turkey. At this site, from the second half of the 10^th^ millennium BC onwards, early Neolithic groups constructed several monumental circular limestone buildings up to 20 m in diameter and with pillars that were up to 5.5 m high and often richly decorated [64]. The buildings have been interpreted as the loci of cultic activities [52, 58, 64], and large-scale work feasts have been discussed as a model to explain the gathering of a workforce large enough for the building activities [51, 64, 72, 73]. Arguments regarding the subsistence of the builders and the likelihood of feasting have so far concentrated very much on hunting and the animal bones found at the site [58, 73–76]. Göbekli Tepe has not played any role in discussions of early cereal use, although the late excavator of the site, Klaus Schmidt, proposed that the necessity to supply food for extensive construction activities could have contributed to a need for reliable food sources, accelerating the process of domestication [64]. The reasons for this contradiction can be found–at least in part–in the problematic nature of direct evidence for cereals on site. Although analysis of macrobotanical remains by R. Neef indicate the presence of wild einkorn (*Triticum* cf. *boeticum/urartu*), wild barley (*Hordeum* cf. *spontaneum*) and possibly wild wheat/rye (*Triticum/Secale*), as well as almonds (*Prunus* sp.) and pistachio (*Pistacia* sp.) at Göbekli Tepe [77], the same study points out that only a conspicuously low amount of carbonized plant remains has been recovered, both in handpicked and in flotation samples. The poor preservation was explained by the large-scale relocation of the sediments the samples were taken from (see below), which would have had a negative impact on the fragile plant remains [77]. They therefore cannot be used to estimate the intensity of plant processing on site. On the other hand, excavations recovered large numbers of grinding tools, but these have not yet been analyzed.

Detailed analysis of the grinding tools from Göbekli Tepe has the potential to add valuable information about plant food processing and feasting at the site and to further understanding of the transformation processes occurring during the Early Neolithic in Upper Mesopotamia. The presence of grinding tools on a site is often used as proof for plant processing in archaeological analyses, but use-wear studies have proposed multiple functions for such tools in the Near East, including processing of meat, animal skin, or minerals [78]. As a first step in our analysis it is necessary to outline the ground stone materials recovered in excavation, and to discuss the functional variation of these. Grinding and pounding equipment, consisting of handstones, grinding bowls (larger basalt boulders deepened by use into the form of bowls, see below), plates and pestles, was documented through 3D-modelling by structure from motion, the surfaces were macro- and microscopically analyzed for use-wear. We used replicas of the equipment identified on site to experimentally grind different materials and establish a reference collection for the identification of the observed traces. Further, phytolith samples taken from the sediments inside and outside buildings at Göbekli Tepe and from grinding stone surfaces allowed us to determine and quantify the presence of plants. To contextualize the results, we assessed the spatial distribution of grinding equipment and identified potential activity areas. Here we report on the results of this interdisciplinary approach that has led for the first time to deeper insights into the role of cereal processing and use at Göbekli Tepe. We argue that collecting and processing cereals was an integral part of the subsistence system of the Early Neolithic groups who erected the megalithic buildings at Göbekli Tepe, that work feasts are a likely model to explain their ability to concentrate the necessary work force at the site, and that providing food (and drink) for these work feasts would have required large-scale food supplies and their processing at certain times of the year.

## Göbekli Tepe

Göbekli Tepe is situated high on the Germuş mountain range at ca. 770 m asl., offering a wide view over the Harran plain to the south. The mound of reddish soil with a height of about 15 m has a diameter of around 300 m and is characterized by several hilltops divided by depressions. It is surrounded by a limestone plateau, which today mostly shows no sediment cover and very scarce vegetation. This must also have been the case during the Neolithic, as numerous quarry sites, cupholes and petroglyphs on the limestone surfaces suggest [77, 79].

**Fig 1:**
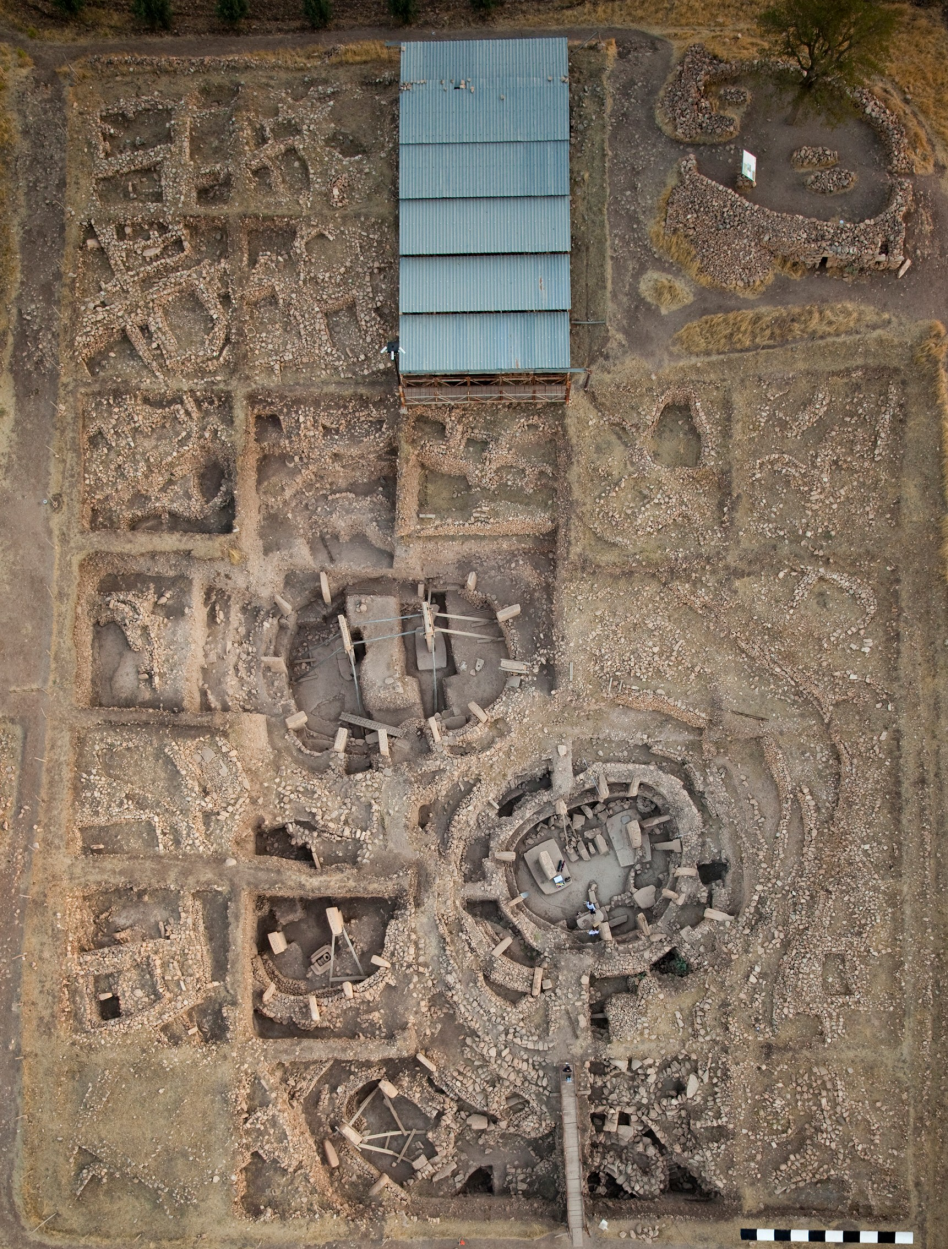
The archaeological site of Göbekli Tepe. Main excavation area with four monumental circular buildings and adjacent rectangular buildings (German Archaeological Institute, photo E. Kücük).

Two types of buildings have been identified at Göbekli Tepe:

(1) Round to oval limestone buildings with inner diameters of 10–20 m, which include T-shaped limestone pillars incorporated into walls conserved to a height of up to 2.5 m (Fig 1) [64]. Bench-like structures run along the inner mantles of the walls. The pillars in the walls stand up to 4 m high and are arranged around two bigger central pillars, reaching 5.5 m. Depictions of arms, hands and clothing on some pillars indicate their anthropomorphic character; many pillars show reliefs of wild animals and abstract symbols, depictions of humans are rare [58, 64].

**Fig 2:**
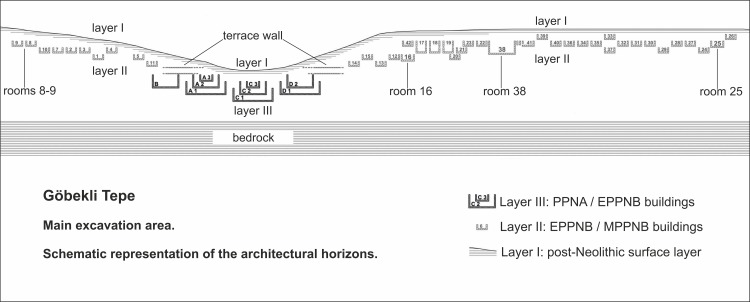
Stratigraphy of Göbekli Tepe. Schematic representation of the architectural horizons in the main excavation area (modified after a plan by D. Kurapkat [56]).

Five such buildings have been excavated in the lower-lying areas of the mound (buildings A-D in the southeastern, building H in the northwestern depression), several more have been detected by georadar [51]. In buildings C and D, the floor level is formed by the artificially smoothed bedrock. The two central pillars stand in pedestals carved from the bedrock as well. Building B has an artificial ‘terrazzo’ floor made of burnt lime and limestone chips; in buildings A and H the floors have not been reached yet. The question of whether the monumental buildings were roofed is still hard to answer [64], but much speaks in favour of the structures having been partly subterranean with entrances through the roofs [56, 80]. During excavations, these structures were identified as belonging to an older layer (III) of site occupation [61, 63, 64] dated to the PPNA [81, 82, 83] (Fig 2).

The buildings are multi-phased and were long-lived [56, 84]. The general tendency, best observed in building C so far, was to consecutively add new circle walls inside the buildings, thus making them ever smaller [56, 84] (Fig 2). Building analysis has highlighted three ring walls for building C [56, 84]. The two outer walls each have three major building phases, the innermost ring has four. Pillars were taken out of the earlier buildings and re-used in the younger phases. The intense construction and rebuilding activities indicate that this building could have been in use not only for several decades, but even centuries [56]. The large round buildings have been described as monumental due to their size and also in comparison to the second type of architecture known from the site.

**Fig 3:**
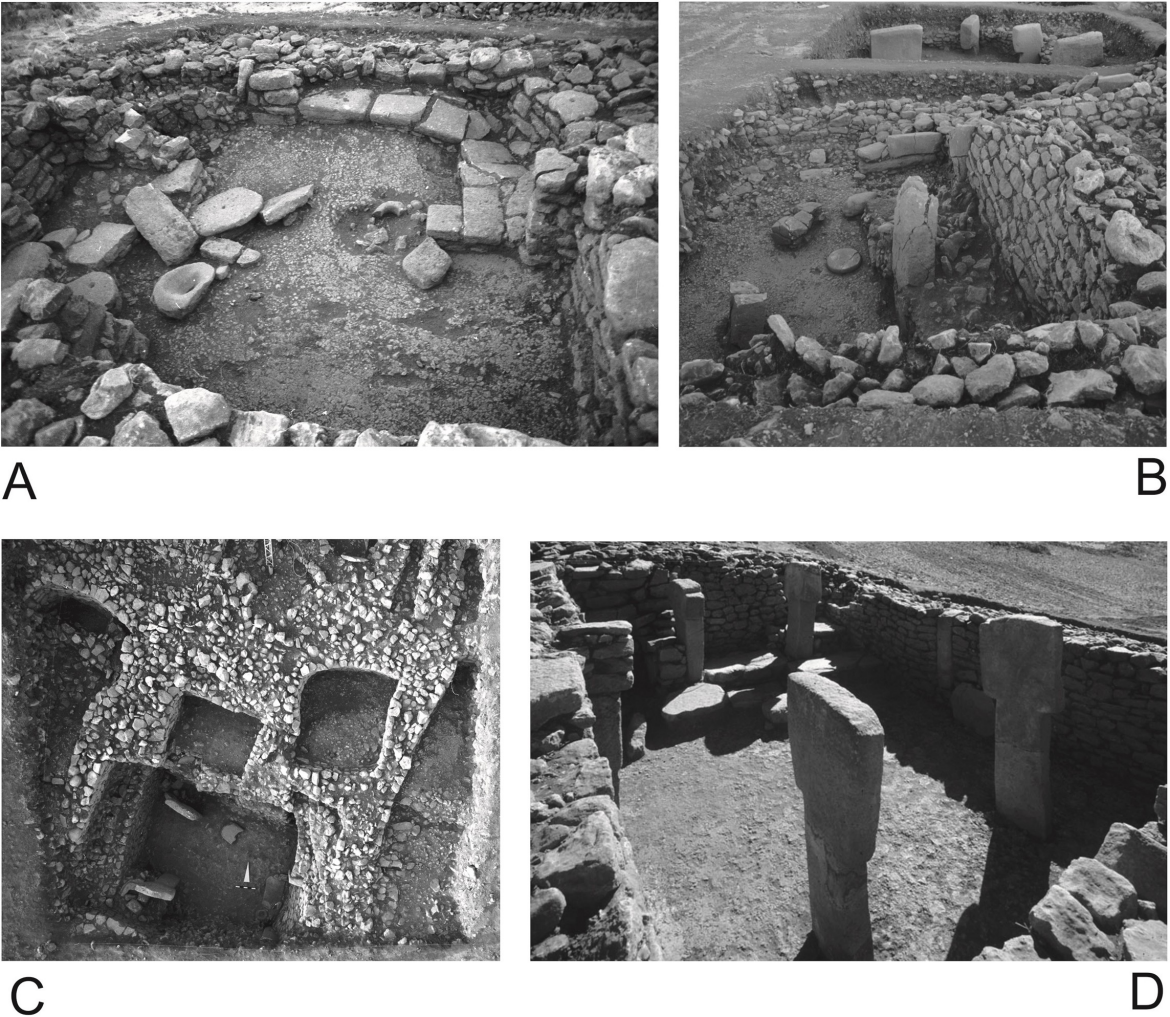
Göbekli Tepe, rectangular buildings of layer II in the main excavation area. (A) Building 25 with grinding bowl on the floor and benches; (B) building 9 with pillars and plate; (C) building 16 with three cell-like adjacent rooms; (D) building 38 (the lion-pillar building (German Archaeological Institute, photos K. Schmidt).

(2) Larger (up to 29 m^2^) and smaller (up to 5 m^2^) rectangular buildings with ‘terrazzo’ floors made of burnt lime and limestone chips. These may have been one-story buildings with entrances through flat roofs [56, 80]. Especially the larger buildings feature up to 2 m high T-shaped pillars, which are, however, no longer positioned in the center of the buildings. These larger buildings were also sometimes fitted with benches and platforms (Fig 3).

During excavations, these buildings were identified as belonging to a partially younger layer which is superimposed on the monumental architecture in some parts of the mound [56, 61, 63, 64], but has mainly been exposed on the higher-lying areas of the site. This layer II was attributed to the early and middle PPNB [64, 81, 83] and has received less attention so far, aside from reconstructions of the building history [56].

**Fig 4:**
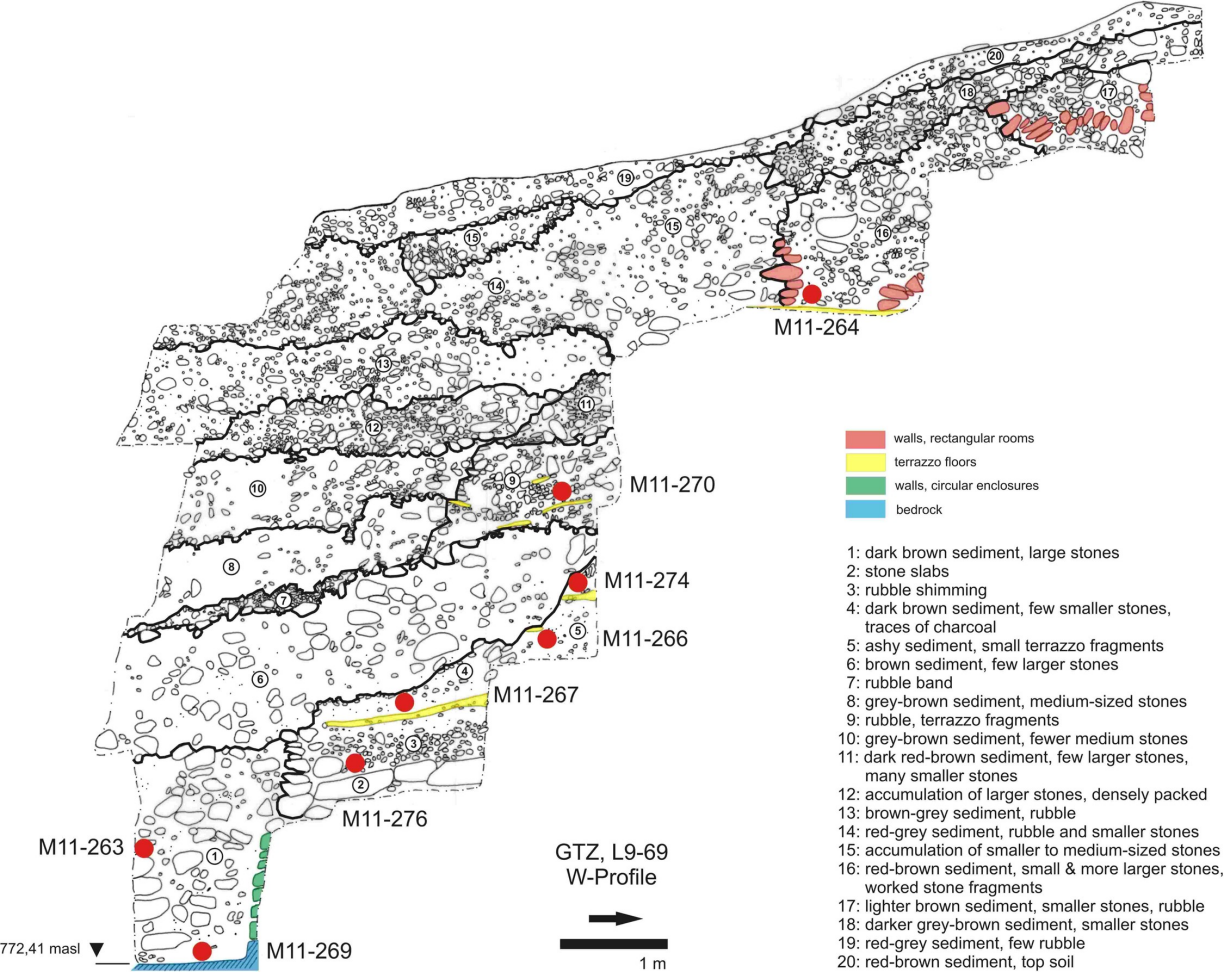
Stratigraphy of Göbekli Tepe. Western profile of area L9-69 in the main excavation area showing several terrazzo floors in layer II and the second ring wall of building D as well as the origin of tested soil samples (German Archaeological Institute, compilation J. Notroff).

D. Kurapkat has shown that the rectangular buildings were constructed immediately next to each other [56]. In some cases the buildings even share walls; as a result there are very few stratigraphical superpositions. Kurapkat views most of the buildings as roughly contemporaneous (Fig 2) [56]. A chronological depth of the rectangular buildings is indicated, however, by sequences of terrazzo floors within them (Fig 4). Unfortunately, in most cases excavations stopped at the uppermost floor level. We thus only know the last phase of use for many layer II constructions. These last phases of use of individual buildings may not belong to one contemporaneous horizon though.

Changes in iconography can be detected between the monumental round buildings and the rectangular buildings. Animal depictions are–with few exceptions–absent in layer II, while there are more ‘arms and hands’ motifs. In addition to the animals and symbols depicted in flat relief, Göbekli Tepe’s buildings, both in layers III and II, have yielded a series of anthropomorphic and zoomorphic sculptures, which repeat the same types canonically (e.g. wild boar with large fangs, snarling predators: [85, 86]). What is absent from both building types is evidence for hearths or fireplaces. Cooking activities seem to have taken place outside the buildings, not leaving identifiable remains behind inside the buildings. Another possibility is that erosion or other processes have destroyed the traces of fire.

**Fig 5:**
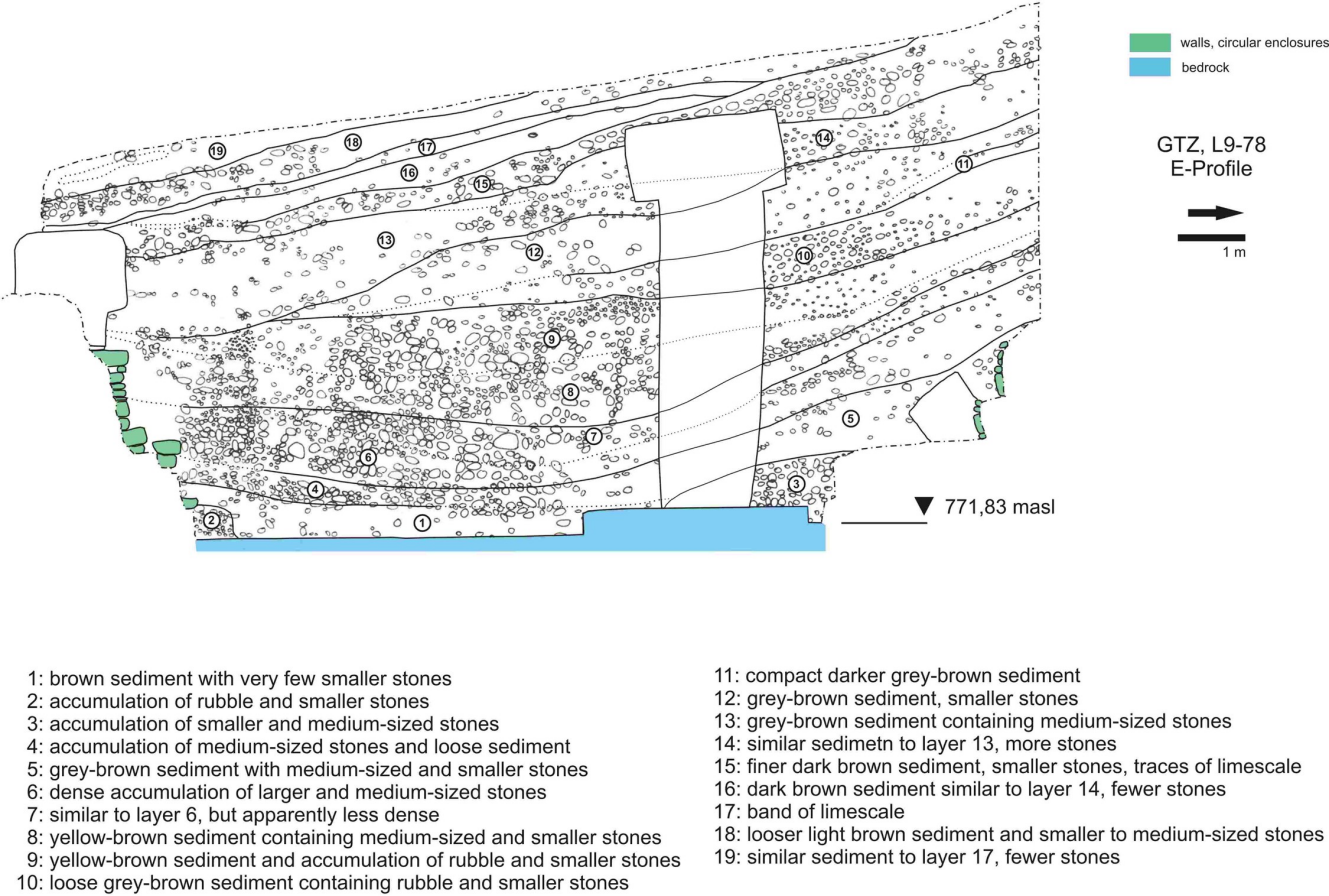
Stratigraphy of Göbekli Tepe. Eastern profile of area L9-78 in the main excavation area, cutting through building D (German Archaeological Institute, compilation J. Notroff).

Recently it has become clear that the latest construction phases of some of the circular buildings may have been still in use up to the Early PPNB and thus layer II [56, 81], while others could already have been refilled at this point. This would imply that some of the rectangular buildings could be identified as residential structures contemporary to the late monumental ‘special’ buildings. There is evidence for acts of backfilling (or intentional burying) at the end of the latters´ respective use-lives (observed during excavations in the lower levels of the monumental buildings [64]), which seem to have included the deliberate deposition of material culture, especially sculptures [49, 85]. Sections through the filling of building D show a relatively leveled stratum immediately above the floor level (Fig 5, layer 1), followed by six units that suggest rapid backfilling from the building´s margins towards the center, resulting in heaped sediments at the walls and a lower thickness in the center (Fig 5, layers 3–8). Two intentionally deposited anthropomorphic limestone heads were discovered near building D´s western central Pillar 31, at the border between units 7 and those below it, further substantiating the case for intentional backfill [49, 85, 86] (Fig 5). The intentional backfilling events were identified as one cause of the bad preservation of charred plant remains [77], which would have been too fragile to withstand the large-scale relocation of sediments.

It is still not entirely clear where the material for the refilling originated from. There is one radiocarbon sample of collagen from an animal tooth from the deepest layer (Fig 5, layer 1) inside building D (KIA-44701, 9800 ±120 14C-BP), resulting in a calibrated age between 9746–8818 cal BC at the 95.4% confidence level [82]. This date has a time-span which is in concordance with an earlier measurement made on clay mortar from the ring wall between Pillars 41 and 42 (KIA-44149, 9984 ± 42 14C-BP, 9745–9314 calBC at the 95.4% confidence level) [82], attesting that PPNA materials were part of the sediments used to repair and backfill the building.

The block of probably intentional backfill is followed by bands of sloped rubble layers, which indicate slips of sediment from higher-lying parts of the mound into the lower-lying buildings as a factor in the final sealing of the building (Fig 5, layers 9–19). Several bands of sediment fill the building up to the top of the walls still preserved today (Fig 5, layers 9–13). Further strata lie above the top of the walls and cover the central pillars (Fig 5, layers 14–19). Judging from the height of the probably intentional backfill, we assume that the contours of the buildings and especially the pillars would have been visible for a longer period of time after the abandonment of the monumental buildings; this may have also been the case for some of the higher pillars in the ring wall. Cup marks on the heads of several pillars hint that people continued to engage with the older structures at the site [64].

**Fig 6:**
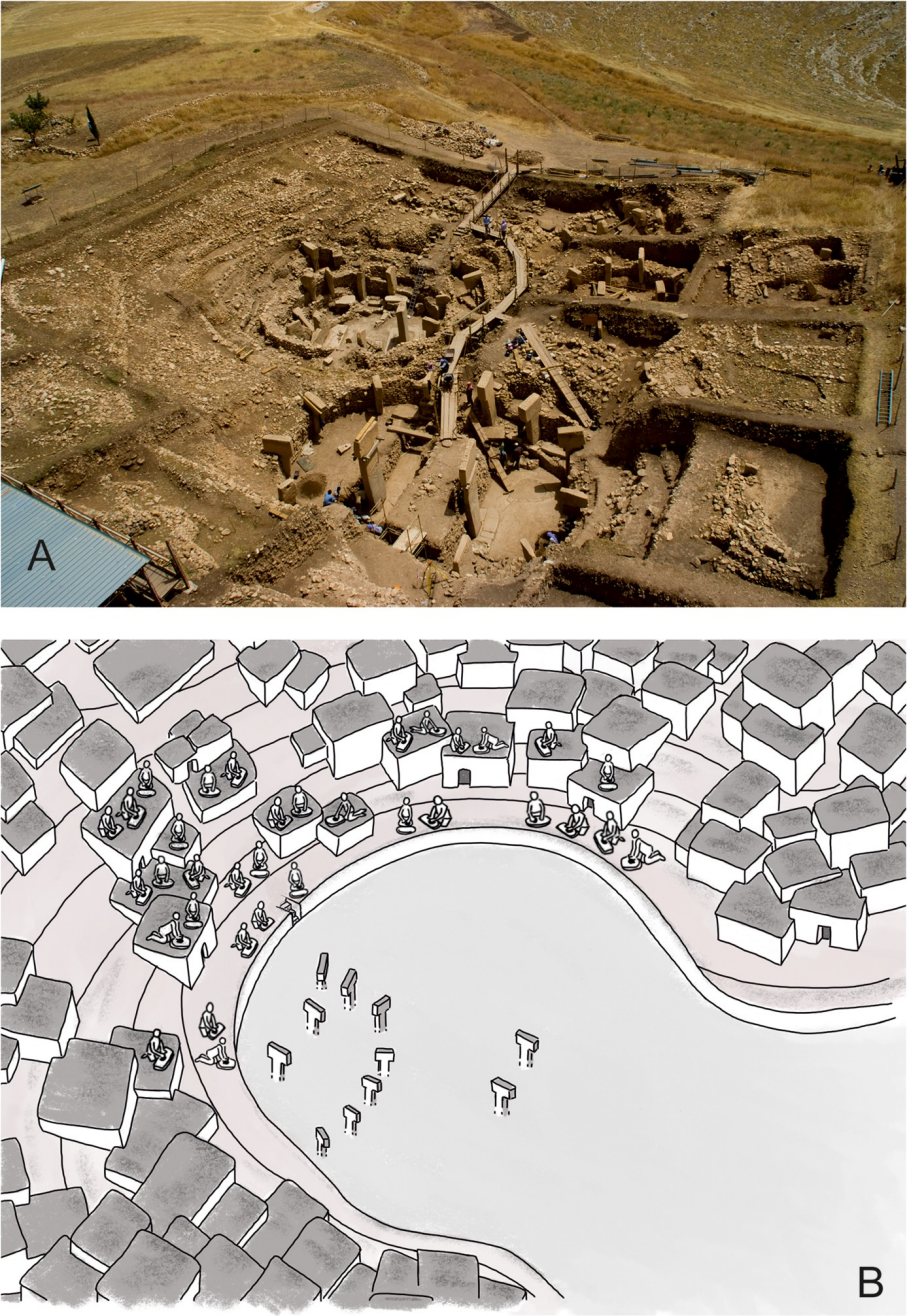
Göbekli Tepe, excavated features and reconstruction drawing. (A) Göbekli Tepe, excavations in the main excavation area, 2010. (B) Reconstruction drawing of communal work on the terraces and roofs with a view towards the monumental buildings (German Archaeological Institute, photo N. Becker, graphic J. Notroff, based on an architectural reconstruction by D. Kurapkat [56]).

A terrace wall encircles the area in which the monumental buildings lie [56, 87]. One of the functions of this wall could have been the prevention of further sediment slips into the monumental buildings. Younger, or in part contemporary, rectangular buildings deliberately spared the round buildings, forming terraces that lined the depression around them (Fig 6). Access to the circular buildings was possible by a stairway included in the terrace wall. It is thus possible that the wall was built when some of the monumental buildings were still in use, i.e. during the period of overlap between round and rectangular buildings.

Site formation processes included phases of rapid accumulation interchanging with periods of inaction and humus formation, as a pedological analysis revealed [83]. One humus layer with a thickness of 20 cm is located at a depth of 1.5 m, superposing layer II above building D in the northern bulk of excavation area L9-68 [83]. A radiocarbon date from this layer revealed an age of 8860+/-60 BP, giving a calibrated interval of 8240–7780 cal BC (95.4% probability) for the last PPN activities at Göbekli Tepe [81, 83]. Layer I is the label for the surface soil. The division into layers III and II was based not only on architectural change. There are some spots where layers II and III clearly overlap stratigraphically [56, 64].

The general distinction between three large stratigraphic ‘blocks’ therefore seems to be correct. However, these blocks span significant periods of time and, as explained above, incorporate many phases of construction and refilling. Whereas building phases have been analyzed for the large monumental buildings and some of the rectangular structures [56, 84], work on the stratigraphy continues and will ultimately lead to a much higher resolution of activities on site. One recent insight regards evidence of the presence of yet another building type: simple C-shaped or oval to round structures, sometimes subdivided by a wall, without other standardized interior fittings. These buildings have been addressed as a fourth layer in some reports [63]; in one excavation area (L9-59) there is a stratigraphic sequence of lower-lying oval structures and superimposed rectangular buildings. The small round buildings may thus be older than, or contemporary to, the monumental structures. As the exact chronology of these structures is still uncertain, we excluded them from our analysis.

## Functional analysis of grinding stones

In archaeological analyses, the functions of grinding equipment are usually assessed by use-wear analysis on original finds and comparison of traces to experimentally obtained reference collections [78]. There has also, however, been a trend to separately evaluate use-wear and surface transformations of objects and their formal development [21, 22]. These two lines of analysis should be brought together for a consistent interpretation of tool functions.

**Fig 7:**
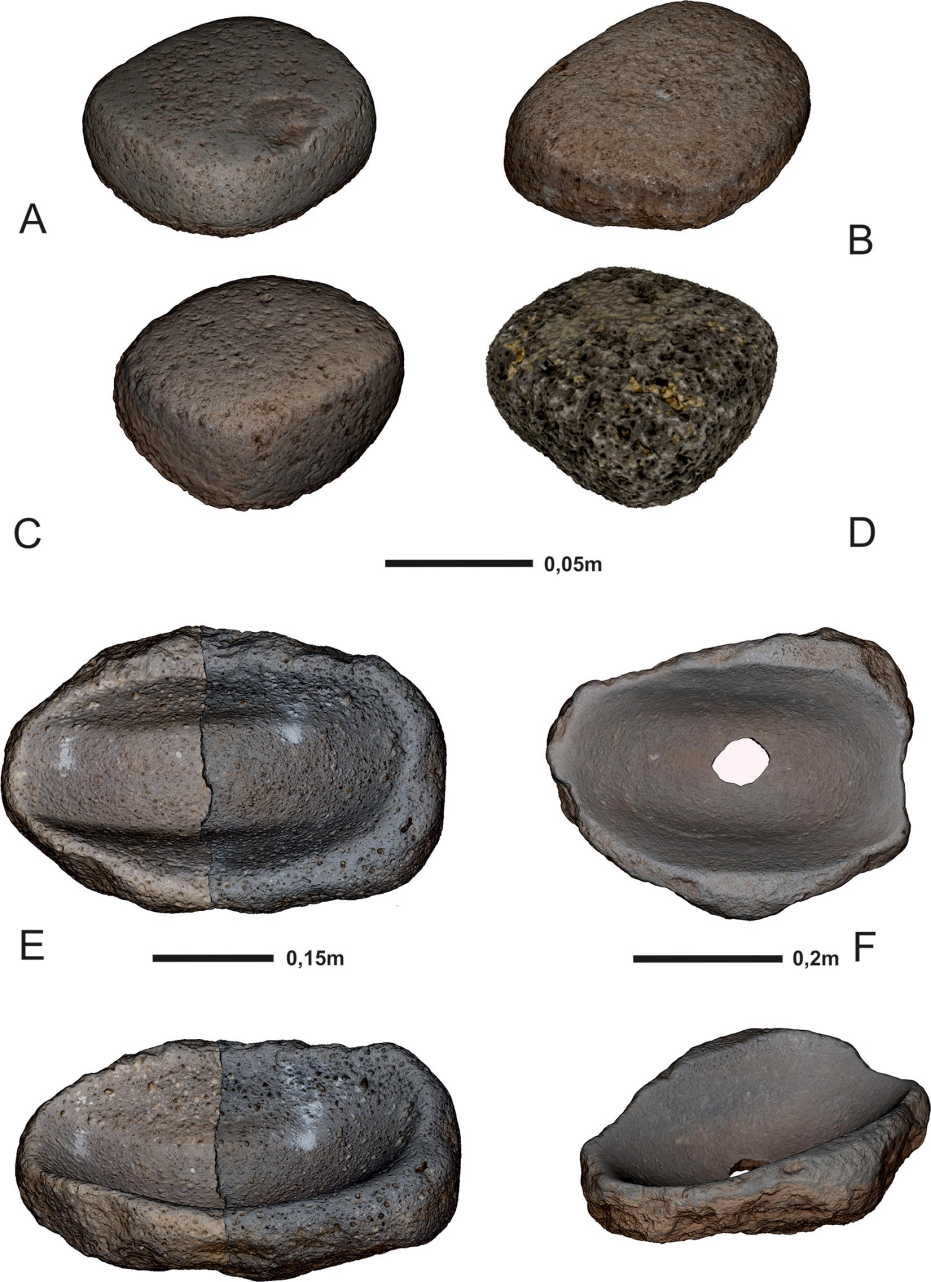
Grinding tools from Göbekli Tepe. (A), (C) Neolithic handstones of type 1; (B) Neolithic handstone of type 2; (D) Experimental handstone of type 1, produced as copy of (C); (E, F) Neolithic grinding bowls (German Archaeological Institute, 3D-models H. Höhler-Brockmann and N. Schäkel).

At Göbekli Tepe, handstones, grinding bowls and plates, mortars and pestles have been discovered in large numbers (Fig 7 and S1–S3 Tables). These tools are made of basalt, which occurs as an outcrop approximately 2 km from the site. As no work at all has been done on grinding tools from Göbekli Tepe so far, we discuss every category separately and in detail with reference to the types occurring on site and the possibility to infer functions from use-wear analysis. Permission for field research was granted by the Ministry of Culture and Tourism of the Republic of Turkey in the frame of the Göbekli Tepe Research project.

**Fig 8:**
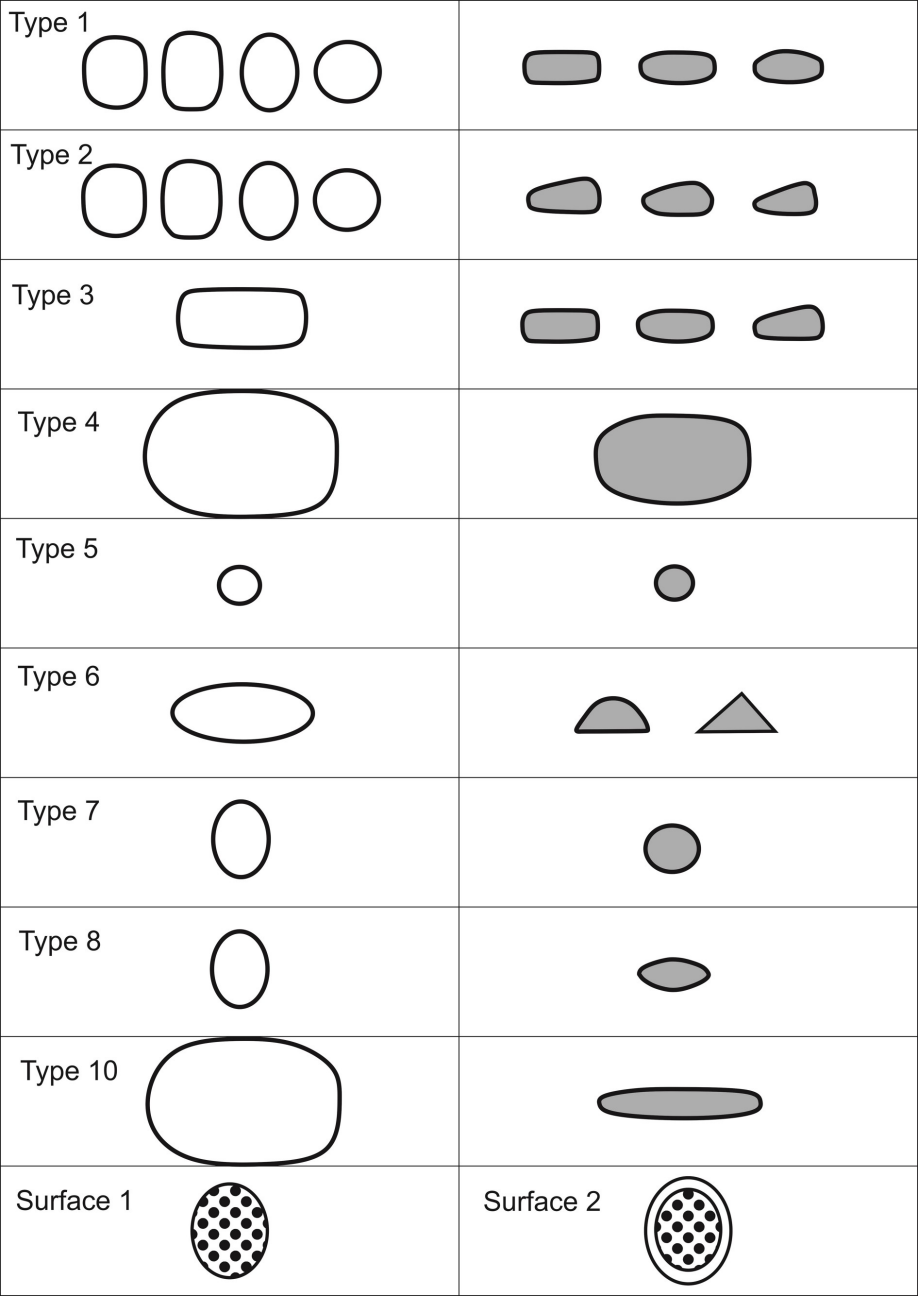
Typology of the handstones from Göbekli Tepe. Schematic depiction of shapes (white), profiles (grey) and surfaces (hatched) of handstones at Göbekli Tepe (drawing L. Dietrich).

Almost 3400 handstones of coarse and middle-coarse basalt were found (S1 Table). They come in a variety of shapes and sections (Fig 8 and Table 1), which is characteristic for this find category [22]. Previous classification systems are based either on shapes [88], separately on shapes and sections [21, 22], or on a combination of both to account for functional and stylistic significance [45]. In some classification systems, the shapes and modification of surfaces were linked to possible handling [88]. Other systems have stressed the chronological significance of some features, like lentil-shaped sections of handstones as a later PPNB feature, resulting from prolonged bidirectional instead of circular abrasion [89]. For the present study, we were interested in describing functional types [90] and variables were chosen accordingly. Shapes define the handling, while sections, sizes and weights are determining factors for grinding motions and thus surface transformations during work processes. Handling and motions define the degree of efficiency and productivity. The outlines are not the main defining variables for types, other than in formerly proposed typologies. A total of ten types of handstones can be differentiated, within the sample of 1166 finds which were analyzed regarding form and use-wear (Fig 8 and Table 1).

**Table 1:** Typology of the handstones from Göbekli Tepe.

Type 1	Oval to subrectangular in topview, high and square or pillow-shaped sections (485 finds).
Type 2	Oval to subrectangular in topview, wedge-shaped in section (256 finds).
Type 3	Elongated subrectangular in topview, pillow- to wedge-shaped sections (4 finds).
Type 4	Broad-oval in topview and in section (16 finds).
Type 5	Small, round, ball-shaped (5 finds).
Type 6	Elongated-ovaloid in topview, d-shaped or triangular in section (120 finds).
Type 7	Oval in topview, round in section (18 finds).
Type 8	Oval in topview, lentil-shaped in section (18 finds).
Type 9	Irregular shape and section (25 finds).
Type 10	Broad-oval in topview, flat-oval in section (51 finds).
Not classifiable due to object preservation	127 finds
Preforms, roughouts, miniatures	43 finds

The actual shape of some types is a result both of preform and change through use. Types 1, 2, 6, 7 and 8 have different sections. Handstones of these types can be used with one hand. Types 1 and 2 are most frequent at Göbekli Tepe and finds include preforms and tools in different stages of use. Types 1 and 2 give the most complete information on the *chaîne opératoire* and the changes in form occurring during use. First, basalt blanks were reduced by pecking to the actual tool form. Types 1 and 2 require a pendular grinding motion due to their high section when they are used bidirectionally. Therefore, the section changes with intensity of use by becoming more convex (starting from near-rectangular for type 1 and wedge-shaped for type 2) and the edges are rounded. Also, their thickness is reduced. The degree of transformation however is not very high, the reduction of thickness between ‘fresh’ and used pieces of types 1 and 2 amounts to a loss of not more than a few centimeters of material. Types 1 and 2 were also used with circular motions, causing a reduction concentrated in the center of the handstones. Types 6, 7 and 8 determine bidirectional motions parallel to the surface. Through use, the sections of these types would become flatter. Types 3, 4 and 10 are large and were probably used with two hands; types 4 and 10 cannot be used for circular motions. The round type 5 cannot be used bidirectionally.

**Fig 9:**
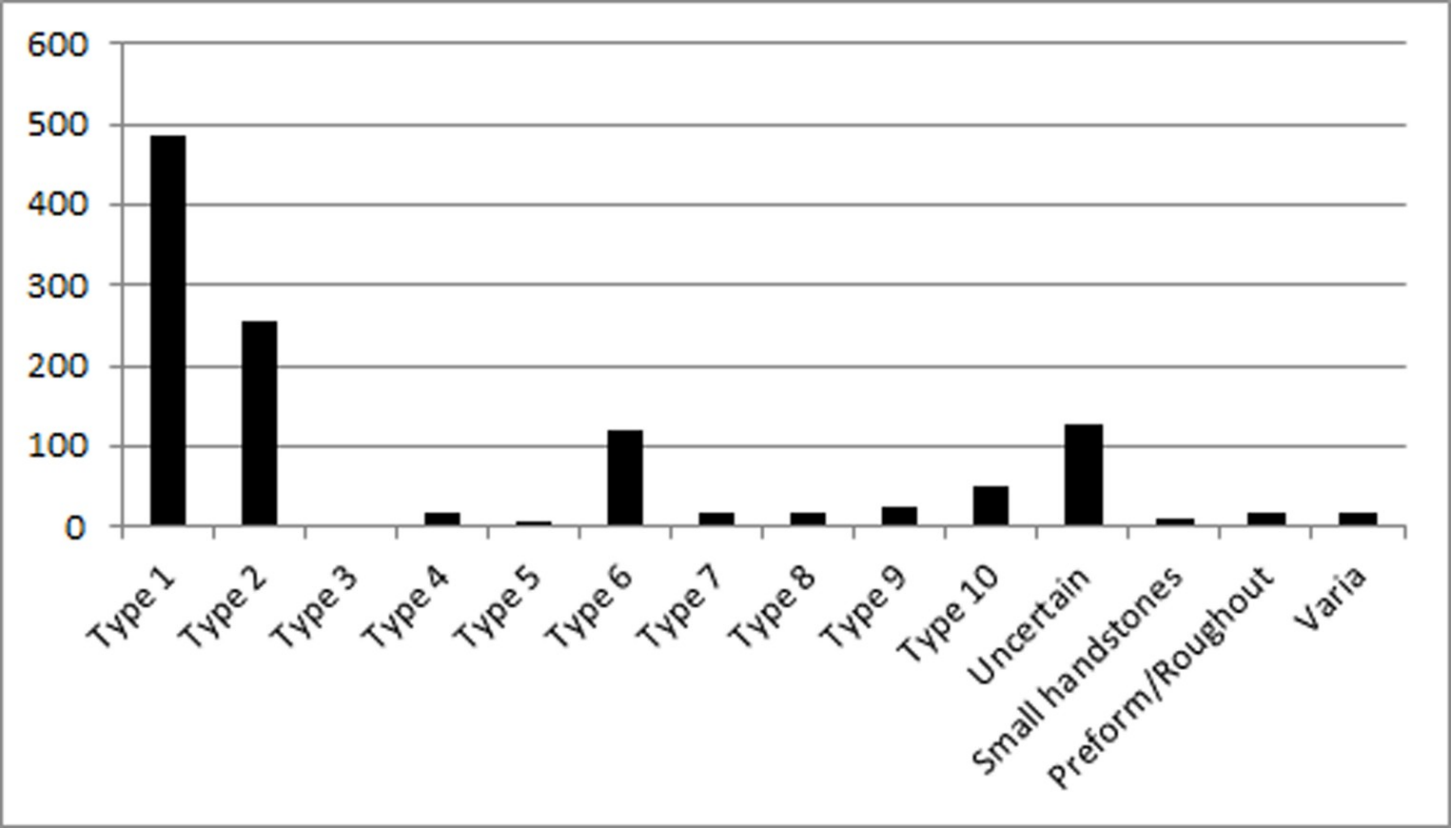
Distribution of the types of handstones at Göbekli Tepe. The statistical analysis is based on 1166 finds, which were analyzed in terms of both formal and functional aspects (graph L. Dietrich; the graph does not include preforms).

The variety of forms seems fairly high at first glance and could hint at a larger spectrum of tasks performed, but types 1 and 2 dominate the tool assemblage with a proportion of 75% (Fig 9). They are the main grinding tools at Göbekli Tepe. Both types were produced in their respective forms intentionally from blanks. Ongoing experimental studies have shown that an average of 1.5 h was needed for the production of one handstone of this type [91]. The relatively simple [91] and rapid production process and the readily available raw material may explain why damaged or fragmented handstones were not repaired but discarded. Only 7% of the 1166 finds which were analyzed regarding form and use-wear were preserved completely.

The use-wear found on types 1 and 2 is very uniform, which also suggests that the tasks performed with these handstones were equally uniform and standardized. The macroscopic analysis of use-wear included optical examination and tactile investigations. The most common surface type is pitted in the center, showing a combination of smaller flat plateaus on the high topography and depressions, and more flattened areas on the margins (surface s2, observed on 251 handstones; Fig 8). On handstones with a wedge-shaped section, the lower part shows more use-wear indicating the handling position. Different sections of handstones of types 1 and 2 with surface 2 indicate different degrees of use, with convex sections which are more worn; these items dominate the find spectrum at Göbekli Tepe. The second common type of working surface is entirely pitted (surface s1, observed on 232 handstones). In the case of handstones of types 1 or 2 with surface 1, flat shapes are more worn than convex ones and are more numerous. Leveled surfaces (surface s3, s4) and surfaces with irregular plateaus (surface s5 and s6) appear less frequently (altogether on 239 finds). A number of finds have large sinter layers or are too fragmented to be determinable (381 finds). 66 handstones show no use-wear. Further, some handstones have multiple working surfaces. A standardized fragmentation pattern could only be observed for handstones of types 1 and 2, with most objects being broken in two.

**Fig 10:**
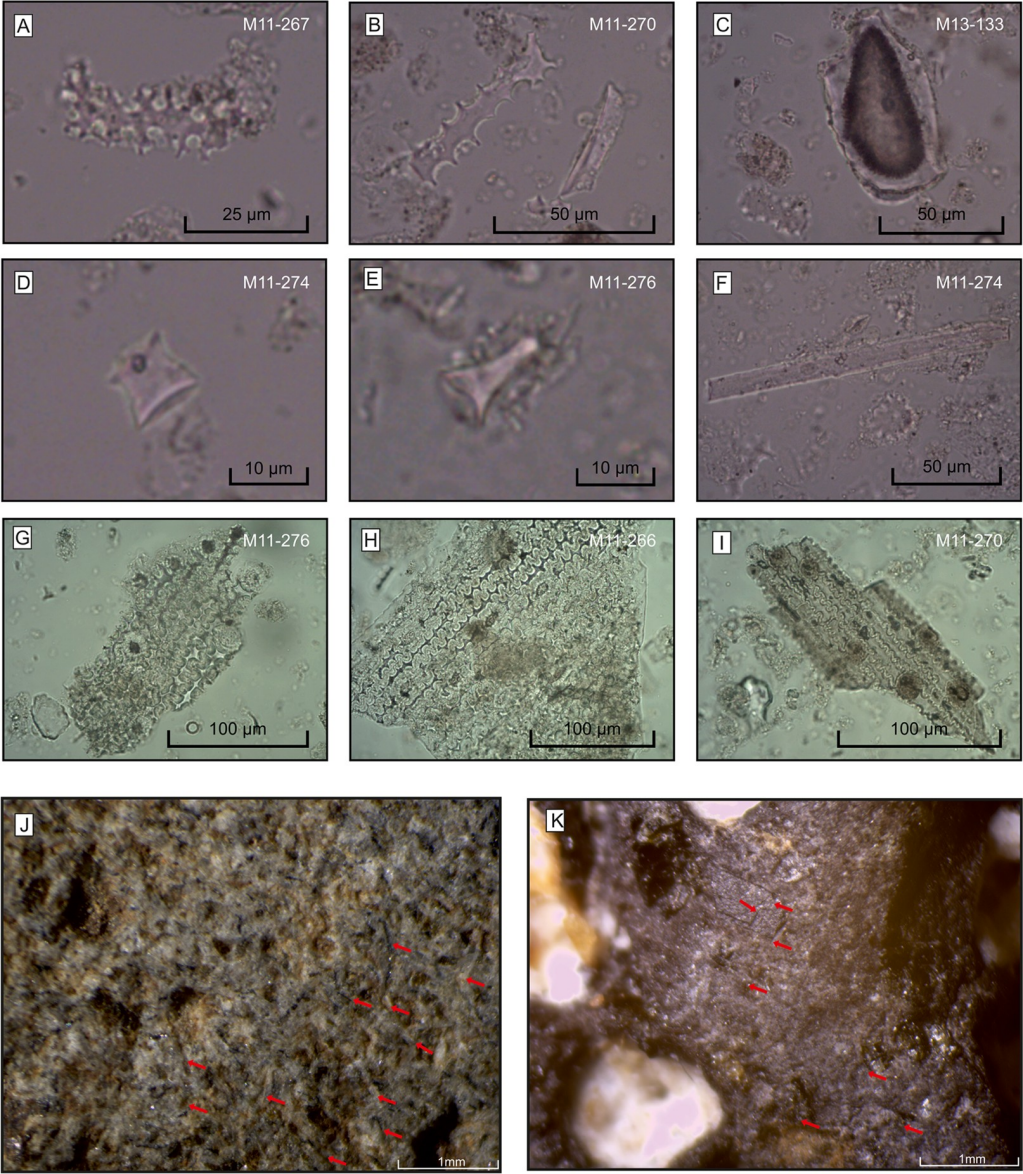
Göbekli Tepe. **Microscopical analyses of phytoliths and use-wear.** (A-I) Photomicrographs of phytoliths identified in the samples (400 x magnification). (A) elongate dendritic; (B) elongate echinate (left) and elongate trapeziform short cell (right); (C) bulliform cell; (D) rondel short cell; (E) tall rondel short cell; F: cilindroid psilate cell; (G-I) multicelled structures from inflorescence bracts (photos J. Meister). (J) Photomicrographs of use-wear from Neolithic handstones and (K) from experimental handstone replica used for processing einkorn (German Archaeological Institute, photos L. Dietrich).

40 handstones with surfaces s1 and s2 were analyzed microscopically, following the procedures proposed by Adams [25, 92, 93] and Dubreuil et al. [78] for the examination of Neolithic grinding stones. The main observable traces are loose agglomerations of short grooves of differing densities and the formation of plateaus on the high parts of the topography (Fig 10J). Plateaus with U- or V-shaped profiles are visible on the high topography of the pitted area in the center of the handstones at low magnifications (x10, x20 and x40). Previous observations link profiles of this shape with the processing of cereals [94]. The plateaus are flatter on the margins of handstones.

To test the functions implied by microscopy for handstones of types 1 and 2, we decided to establish a reference collection of use-wear traces. The experimental program was primarily designed to explore the tribological wear of handstone surfaces through mechanical abrasion with different materials, as this is the best method to recognize their functions [25, 78, 93, 94]. As we wanted to examine the wear formation and to compare the wear patterns, and not the process of the stones’ abrasion [78, 93], the experimental programs were conducted manually. Next to stone and material characteristics [25, 93], we considered form and weights of the processing tools as important parameters in wear formation. Therefore, we produced and used exact replicas of the Neolithic handstones. Both form and weights of the replicas as well as the used surfaces of the archaeological specimens thus would reflect the last step in the object biographies. Previous works highlighted the processing of cereals, drupes, and meat as main foodstuffs of the Neolithic in the Near East [17–20; 94; summarized in 78]. The concrete framework of the experiment was derived from the results of macrobotanical analyses [77] and phytoliths at Göbekli Tepe (see below), indicating the presence of wild einkorn and barley, almonds and pistachio, as well as from the archaeozoological data on the consumption of meat [73–76]. Therefore, we used einkorn, almonds and raw red meat as objects for experimental analyses. We choose a group of six persons aged between 20 and 40, including women and men, to conduct experiments lasting more than ten hours at various times of the day and different temperatures over a period of one year with the task to produce fine and coarse flour. We documented traces on the surfaces of the tools macro- and microscopically at regular intervals. Documentation included descriptions of movements, correlation between amounts of processed foodstuffs and necessary times for grinding, and correlation between weights of grain and obtained product. Modifications of the surface were registered after macroscopical and microscopical examinations. We examined two spots on the grinding stones used in the experiments, one in the center and one at the margin of the working surface, at magnifications of x10, x20, x40, x80 and x160. We observed that results can be significantly influenced by the spots chosen for microscopical analysis within the areas with traces.

The results of the einkorn set revealed similar grooves and plateaus to those described above (Fig 10K). Handstones with rectangular or trapezoid sections both require a pendular bidirectional grinding motion (S1 Movie), which is appropriate for the processing of cereals and possibly also pulses; these stones are difficult to use for processing nuts and meat. Soft nuts require circular grinding movements best achieved with pestles. Handstones with the weight range observed in types 1 and 2 are too heavy for the processing of nuts. Grinding nuts on coarse basalt leads to considerable losses. Meat fibers have to be broken with pounders; handstones are not appropriate for this task.

The shape of the handstones´ surfaces means that grain collects in the center of the stone, the main forces work on the margins (S1 Movie). The result of continued use is working surface 2 with flat margins and a pitted center. Due to the pressure, the grains are usually crushed to fine flour. Handstones of type 1 or 2 that show surface 2 are therefore diagnostic for cereal processing. Circular or short bidirectional motions, on the other hand, produce less pressure and result in the entirely pitted surface 1. Circular motions were probably used for processing softer cereals or grain polishing. Ongoing experiments indicate that this technique is suitable for crushing malt, which is softer and does not have to be ground to flour (S2 Movie). Tentative evidence has been published suggesting beer brewing at Göbekli Tepe [51]; the presence of surface 1 could be taken into consideration as further evidence of the production of malt and beer.

In conclusion, we were able to experimentally verify the link between microscopically visible loose agglomerations of short grooves of differing densities and plateaus with U- or V-shaped profiles on the high topography of grinding stones and the processing of cereals as proposed by L. Dubreuil [94]. We further observed that the shape and weight of handstones determine the motion and the resulting traces, as they force the worker to adopt certain standardized patterns of movement when grinding material of a certain texture and hardness. The exact replication of shapes and weights of active grinding implements should be considered in further experimental programs for establishing of reference collections. 3D modeling through structure from motion allows a good visualization of the shape and an exact calculation of volumes, which objectify the replication process. As a result, within our sample, assessing the shape and section and macroscopically visible traces can determine the use of grinding equipment in many cases, while microscopic observations support such findings.

Handstones are tools diagnostic for certain tasks, which is not the case with grinding bowls, plates and mortars. In Göbekli Tepe, grinding stones are not standardized, and they usually show multiple uses or are highly fragmented. Grinding bowls (or ‘troughed slabs’ in Davis´ [45] terminology) are larger basalt boulders up to 50 cm in length (Fig 7E) which were deepened by use through both bidirectional and circular motions (as the wear patterns on their walls show–Fig 7E and 7F) into the shape of bowls. Their surfaces are mostly circular and oval, but the actual shape mirrors only the last use. The presence of scars suggests that people utilized pounders to process food here in addition to handstones. This could indicate that other materials besides cereals were also processed in the grinding bowls; there is evidence of ochre on a few of them (see below). A special type of grinding bowl has a hole in its bottom (Fig 7F), which was intentionally made after a period of use, as the striking negatives show. Four finds of this type are known so far. Scars on their margins and their placement on roofs (see below) hint that they were used for dehusking cereals. Grinding plates are thin (up to 10 cm) compared to the grinding bowls and were also used both with bidirectional and circular movements. Only a few examples have been found though. Additionally, massive mortars with small round working surfaces (up to 12 cm diameter, on average 6 cm deep) have been found. They were used in combination with pestles and show traces of circular motions on their walls. Their use in cereal processing has not yet been experimentally tested. Pestles from Göbekli Tepe have highly fragmented working surfaces that do not easily permit use-wear analyses. Studies on materials from other Neolithic sites, however, attest the use of mortars in cereal processing (e.g. [31]).

The use-wear studies show that cereal processing was the most important task performed with a large proportion of the grinding equipment from Göbekli Tepe. However, despite the soundness of this evidence, we chose phytolith analysis as a further approach to prove cereal use at the site due to the absence of charred plant remains. We took samples from the different strata to verify the presence of cereals in different stratigraphical units and give an impression of the intensity of this presence. Samples from grinding stone surfaces were analyzed to further substantiate their connection to cereal processing.

## Phytolith analysis

In a first step, we conducted phytolith analyses on nine soil samples (S4–S8 Tables). Eight of the soil samples were taken from the major N-S profile in the main excavation area (Fig 4). At the moment of sampling, the highest still standing part of this profile, including the whole sequence through building D´s filling sediments and layer II constructions up to the original topsoil, was located in excavation area L9-69, where a deep sounding had been dug to the bedrock [95]. We obtained samples M11-266, M11-267, M11-270 and M11-274 from an agglomeration of terrazzo floors and from open spaces between the rectangular buildings and the monumental building; while sample M11-264 is from the inside of a rectangular layer II building. Samples M11-263 and M11-269 are from building D´s filling (likely from an older building phase, we took the sample between the youngest and an older ring wall, see below); M11-276 is from adjacent stone slabs. As stated above, the younger phases of the monumental buildings are likely contemporary with the rectangular buildings; samples M11-264, M11-266, M11-267, M11-270 and M11-274 stem from contexts possibly dating to this phase; all but M11-264 and M11-267 were taken on remains of terrazzo floors that probably were affected by slope slides into the monumental building. M13-133 is from a different context, a large limestone vessel in a rectangular building at the northwestern hilltop [51] and was mainly included to test phytolith preservation throughout the whole site.

**Fig 11:**
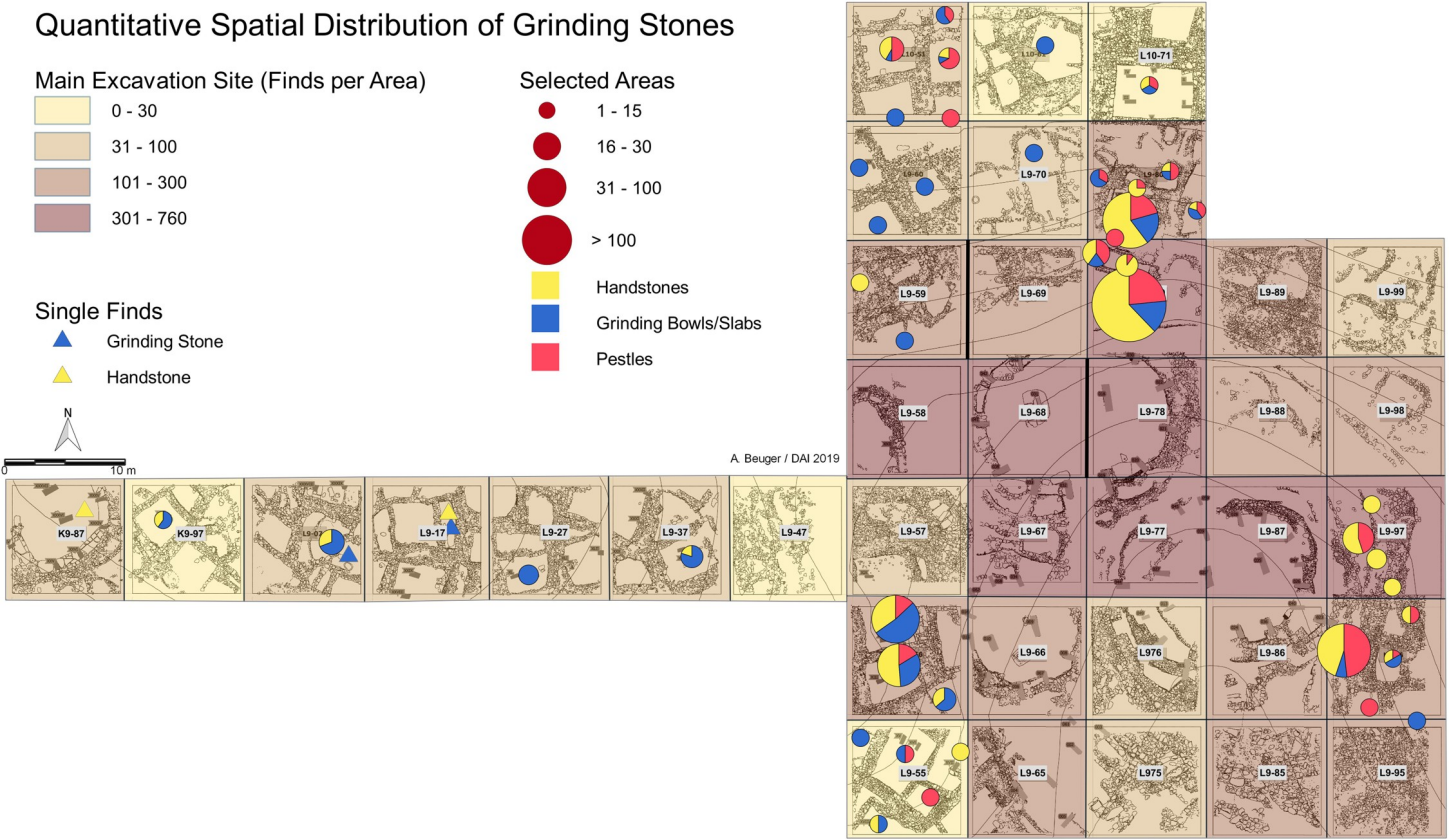
Göbekli Tepe. **Main excavation area.** Distribution of grinding stones (German Archaeological Institute, map L. Dietrich and A. Beuger). The phytolith analyses of grinding stone surfaces were performed on the single finds marked with triangles.

In order to compare the phytolith records of the archaeological sediments with those of selected grinding stones, we took and analyzed a total of fifteen samples from four grinding stones (GS-1 to GS-4; including two handstones type 1, one grinding slab and one grinding bowl) from three different archaeological contexts (S9 Table and Fig 11 for find locations). Samples belonging to GS-2 and GS-3 were obtained from a handstone and grinding slab fragment from a rectangular building (building 125) belonging to layer II, found in a floor layer. We took sample GS-1 from a handstone from the filling of building F [96], and GS-4 from a grinding bowl fragment from building 134 on the floor level, dating to layer II. Since the grinding stones could not be sampled *in situ*, each stone was sampled both by dry and wet brushing, assuming that the majority of phytoliths extracted from the pores of the stones by washing with distilled water were produced during the processing of plant food. In order to obtain control samples, we sampled not only the grinding sides but also the break sides and back sides of the stones wherever possible.

Phytolith extraction of the nine soil samples followed the procedures outlined by Albert et al. [97], while the samples from the grinding stones were prepared according to Katz et al. [98] due to the small amount of sample material. The counting was performed using a Leica DM 2000 microscope at 400x magnification. A minimum of 290 phytoliths with recognizable morphologies were counted per soil sample, while at least 150 visual fields were considered in the counting of the grinding stone samples. Unidentifiable phytoliths were counted and recorded as weathered morphotypes. To allow quantitative comparisons between the samples, we estimated phytolith numbers per gram of sediment by relating phytolith amounts and weights of the processed sample material to the initial sample weights. Morphological identification of phytoliths was based on standard literature [99–104] as well as on modern plant reference collections from the Mediterranean area [105–107]. The International Code for Phytolith Nomenclature was followed where possible [108]. Images of selected phytoliths were recorded using a microscope camera, Optika OPTIKAM PRO 5 digital. Preliminary morphometric analyses of the images were then performed using the ImageJ software with the Phytoliths_plugin [109]. A total of twenty-one morphometric parameters relative to size and shape for seventy elongate dendritic phytoliths, which are unique to inflorescence bracts, were measured for four selected samples (S5–S8 Tables). We obtained descriptive statistics of the means, range, and standard deviations for the parameter largest width for each sample and compared these to the morphometric results obtained from selected wheat and barley species by Ball et al. [110].

Phytoliths were abundant in all nine soil samples examined, ranging from 0.5 to 3.0 million phytoliths per gram of sediment (S4 Table). Overall, the low proportions of weathered phytoliths (mean = 1.8%, σ = 0.8%, n = 9), together with the presence of multicell phytoliths in all samples, indicate that the assemblages are well-preserved. The morphological analyses show that all samples are similar in their morphotype assemblages (S4 and S10 Tables). Grass phytoliths, occurring at a rate of about 81% (σ = 5.3%, n = 9), were the most common group identified. With an average value of 12% (σ = 2.7, n = 9), the amounts of dicotyledonous phytoliths are generally low. For instance, parallelepipedal scabrate phytoliths, one of the most common wood/bark morphotypes, account for only 2.6% at average. We observed other diagnostic dicotyledonous morphotypes such as globulars, polyhedrals or jigsaw-shaped phytoliths in low quantities. Grass phytoliths were divided into the different anatomical plant parts in which they were formed (S4 Table). Short cells, commonly produced in leafs, stems and inflorescences, were abundant in all samples, averaging 25% (σ = 13%, n = 9). According to their morphologies, grasses belong mostly to the C3 Pooid subfamily which includes major cereals (Fig 8D). Tall rondel (‘tower’) short cell morphotypes (Fig 8E), commonly produced in the *Hordeum* genus [111], were noted in almost all samples. Epidermal cells from grass leaves and stems, including, for instance, prickles and bulliform cells (Fig 8C), were observed in all the samples with an average amount of c. 22% (σ = 5.3%, n = 9). Additionally, grass phytoliths derived from their floral parts were abundantly noted in most of the samples (mean = 47%, σ = 13%, n = 9). Inflorescences were characterized mainly by decorated elongate dendritic and elongate echinate cells (Fig 8A and 8B). We observed multicell phytoliths (Fig 8G and 8I) in all samples, although in different proportions (from 0.9 to 15.2% of all the counted morphotypes). They commonly derived from the husks and culms of Pooids, including *Triticum* sp. and *Hordeum* sp. Particularly small numbers of multicell forms could be attributed to the mechanical destruction of phytoliths by grinding, with a variety of deposition and post-deposition processes that could have contributed to this [111]. The sediments inside the rectangular buildings largely contain markers for the upper and middle part of plants. This could be indicative of harvested cereals, as plants are usually collected and transported in sheaves. Discerning between different wild and domesticated wheat and barley species by morphometric analysis of phytoliths is a challenging task [110]. The morphometric means of elongate dendritic phytoliths of four of the samples examined were in line with available morphometric data [110] and could indicate the presence of *T*. *monococcum* (samples M11-269, M11-270), *H*. *spontaneum* (sample M11-133) and *H*. *vulgare* (sample M11-133; S5–S8 Tables), both in layer II and layer III structures. This contrasts with earlier studies, which emphasized that no domestication markers were visible at Göbekli Tepe and should be checked by further sampling.

The observation that phytolith amounts in wet-brushed stone surface samples are usually higher than in the corresponding dry-brushed samples (S9 Table) supports the hypothesis that the sediments extracted from the pores of the grinding stones contain old phytolith assemblages. With concentrations of 92000–391000 phytoliths per gram of sediment, the samples from the grinding sides contain about twice to three times as many phytoliths as the samples from the break sides and back sides of the respective grinding stones (c. 28000–166000 after wet brushing of GS-1, GS-3, GS-4; S9 Table), indicating that they were used for processing plant material. According to the morphological results, grasses dominate the phytolith records of the grinding stones, pointing to the processing of cereals (S11 Table). The highest phytolith density was observed for the handstone GS-1 of type 1 with surface s2, which also supports the results of the use-wear analysis according to which this type is diagnostic for cereal processing. Moreover, multicell phytoliths are totally absent, which could be a result of both dehusking and grinding processes [111]. This pattern has also been observed in grinding equipment from other archaeological sites, where anatomically connected phytoliths were scarce or absent [105, 111, 112].

## Grinding stones in context

Approximately one third of the grinding equipment is from the uppermost layer I and thus chronologically undiagnostic (S2 Table). Nevertheless, the spatial distribution of the remaining finds (Fig 11) is indicative of the organization of cereal processing activities at Göbekli Tepe. We will start our overview with the partly younger layer II and then contrast the findings with evidence from layer III.

## Grinding stones and the rectangular buildings

To facilitate research on the spatial distribution of grinding equipment, we divided the built spaces of layer II into seven zones (S2 Table). Finds on and immediately above floor levels of the rectangular buildings or on floor levels of niches in the buildings (zones 3–5) are considered *in situ*. For the other zones, including the infill (zone 2) and spaces between rooms (zone 7), dynamic and secondary formation processes have to be considered. Zone 6 refers to grinding stones used in secondary contexts as wall stones; zone 1 is the disturbed uppermost part of the buildings´ fillings and the plough horizon.

**Fig 12:**
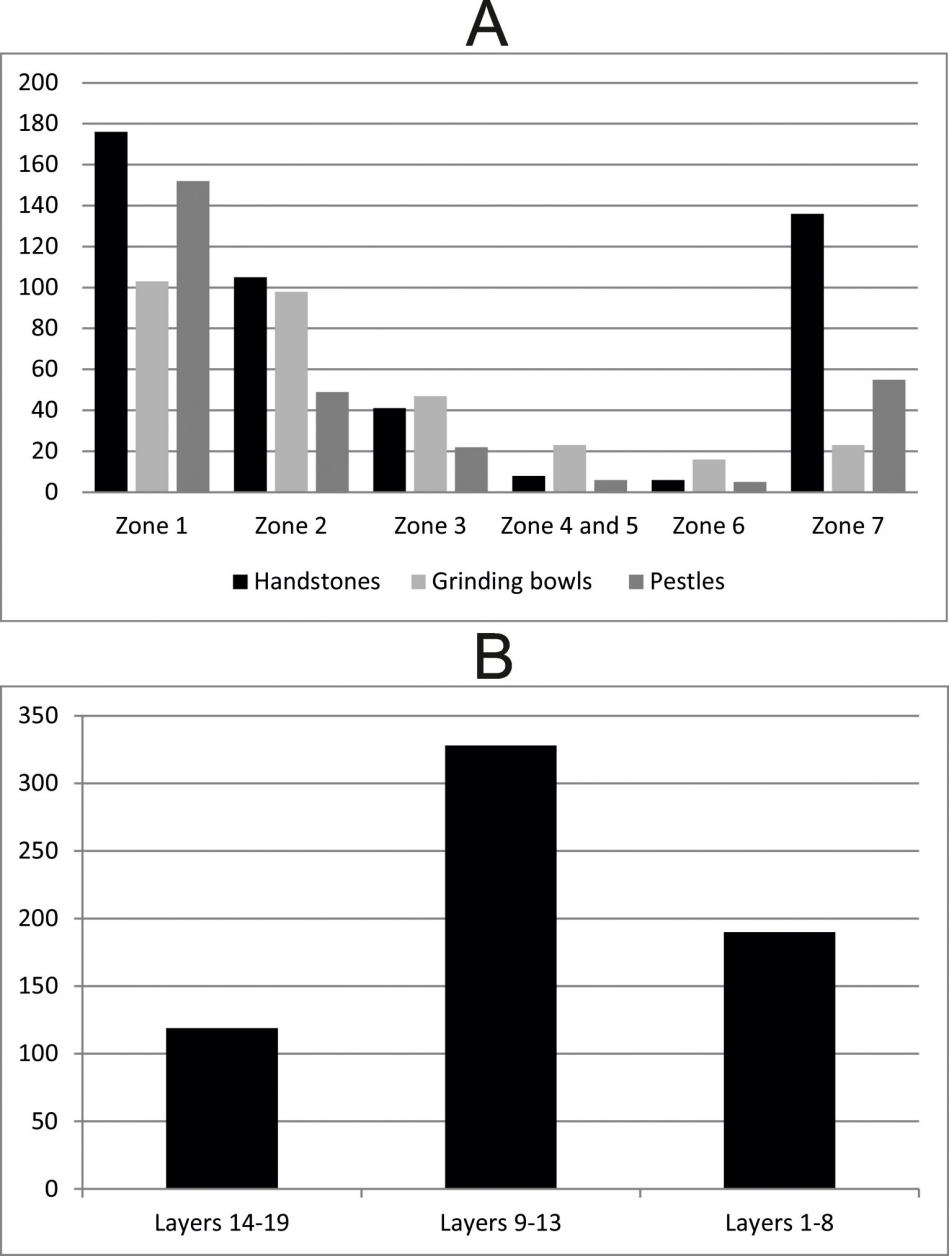
Göbekli Tepe. **Distribution of the grinding stones.** (A) In the rectangular buildings and (B) in the fill of building D (graphs by L. Dietrich).

Generally, layer II buildings contain up to 15 grinding stones within their fills, with up to four on the floor levels (zones 3–5; Figs 11 and 12 and S3 Table). Half of all the grinding stones discovered inside buildings come from the uppermost filling and the plough horizon (zone 1), with proportions varying for different types of artifacts: 36% of grinding bowls and mortars were found in these positions, while 65% of pestles come from zone 1. A further 30% of grinding equipment was found within zone 2 (upper and middle parts of the building fillings), the proportions of grinding bowls / mortars and pestles was nearly equal here. Only 4% of the total number of grinding tools were found on floor levels (zones 4 and 5), with more grinding stones and mortars than handstones and pestles. 13% of the grinding tools stem from the filling immediately above the floor (zone 3) (S2 Table). While these general observations–a low percentage of *in situ* finds and differences in the stratigraphical distribution of different object categories–hold true for all buildings, the composition and thickness of fillings differ, which could imply heterogeneous formation / refilling processes. A sample of buildings (7–9, 25, 16 and 38, for locations compare Fig 2) with well-preserved fillings from different parts of the main excavation area is thus discussed in detail here to check the distribution pattern and further contextualize the grinding equipment (S3 Table).

Building 9 lies on the southwestern slope above building B (Fig 3B). The building measures 5.80 m x 3.60 m with walls preserved up to a height of 2 m and has four pillars. The stratigraphical analysis revealed evidence of four rebuilding phases and relatively fast refilling after the end of use [56]. 46 grinding stones were found within the building fill. Only three complete handstones of type 1 and a completely preserved stone plate were found on the floor, the plate in front of two of the pillars. Seven fragments of grinding bowls and a fragmented pestle were found in the fill above the floor. Most of the other grinding stones, all of them fragmented, come from the middle and the uppermost part of the building fill, which includes roof collapse and wall erosion. Two adjacent buildings are described here mostly to illustrate the agglutinating style of buildings in layer II, although both have only been partly excavated so far. Building 8, which shares a common wall with building 9, has a similar shape and size, but (at the current incomplete state of excavations) only one pillar. Most grinding stones again come from the middle and upper part of the filling. One complete handstone was found on the floor next to the pillar. This hints at a similar situation as observed in building 9. The adjacent building to the south, building 7, has a common wall with building 8. One complete handstone was found on its floor. A similar general distribution of grinding tools within the infill zones was noticeable in buildings on the southwestern hilltop (S3 Table and Fig 11), where the agglutinating rectangular architecture from the main excavation area continues (Fig 1). In buildings 5 and 17, for example, complete handstones and grinding bowls were discovered on the floors next to the pillars, while other grinding stones are concentrated in the middle and upper parts of the filling.

Building 25 lies high on the northwestern slope to the northwest of building D in a cluster of apparently contemporaneously used buildings [56] (Figs 2 and 3A). It is only slightly rectangular with dimensions of 4.20 m x 3.60 m and walls preserved up to 1 m height. All 12 grinding stones, except a grinding bowl, were found in the middle and upper parts of the fill within roof collapse and wall erosion. The grinding bowl was found on the floor near a wall protrusion made of worked stones, a construction which sometimes replaces pillars in Göbekli Tepe´s buildings [56]. Most of the buildings on the northern slope show the same distribution, although their fillings are not similarly well preserved.

Two buildings seem to deviate from the pattern observed so far. Building 16 lies north of building D. It has four pillars, walls preserved up to a height of 2 m, measures 3.8 m x 3.6 m and is again part of a cluster of partially contemporary, partially later constructed smaller, up to 2 m long, buildings [56] (Fig 2). 63 grinding stones were found inside the building, together with the small adjacent buildings the number of finds rises to more than 100. The fill shows consecutive layers of erosion and collapse. Grinding stones were found both in the middle and lower parts of the fill; a concentration was observed in the last 50 cm of fill above the floor. A grinding bowl and a stone vessel were found on the floor next to one of the pillars. Almost all types of handstones as well as some blanks for the production of handstones were present. It seems that this building had a special function either regarding grinding or the production of grinding stones, like a workshop. In any case, the massive presence of grinding stones within the last 50 cm of sediments above floor level indicates the long-term specialized use of the building.

Building 16 is one of the largest completely excavated. But the size and amount of infill do not account for the amount of grinding equipment recovered, as a comparison with the even larger building 38, the so-called lion-pillars building [56, 64], shows. Building 38 is situated on the highest point of the northern slope to the north of building 16, has four freestanding pillars and two more that are incorporated into the walls (Fig 3D). Two of the freestanding pillars have reliefs of jumping wild cats. The building measures 6.6 m x 4.4 m, its walls are still up to 2.10 m high. It shows several successive rebuilding phases, which have already been analyzed in detail elsewhere [56]. There are only three grinding stones in the upper fill from this large and long-used building. The lack of grinding stones may be explained by the special function of the building, indicated by its decorated pillars which are among the very few examples from layer II that show images in the fashion of the older pillars from the monumental buildings [56, 64].

To sum up, although many rectangular buildings show similarities, there are deviations, both with significantly higher and lower numbers of finds. Many buildings are on the same stratigraphical level but have multiple floor levels. In numerous cases, excavations have stopped at the uppermost floor level. Establishing the contemporaneity of floor levels between several buildings is difficult, which hinders estimating the overall number of grinding stones used at the site at a given moment. Further, building interiors are not the only, and possibly not even the most important, locations for grinding cereals. Outside the buildings a large quantity of grinding stones, especially handstones and pestles, were found in the open areas (zone 7) between the square buildings and the circular buildings (Fig 11). The distribution analysis within buildings shows that most grinding stones were in the upper layers of infill, in some cases above roof collapse, and indicates that grinding and processing of cereals most probably took place on the roofs in addition to the outside areas. Especially light grinding bowls with holes (Fig 7F) are of interest as they were found ‘fallen’ in four cases, lying upside down in the upper fills, indicating that they had most probably originally been placed on flat roofs. The stereotype finds of complete grinding stones (especially handstones) on floor level, specially positioned next to the pillars could, in contrast, represent the intentional deposition of these stones at the moment a building was abandoned, as other *in situ* finds are conspicuously scarce on floor levels. Göbekli Tepe has produced clear evidence of the intentional deposition of other items of material culture, especially of sculptures and relief fragments [49, 85].

If the rooftops and open spaces on the terraces around the low-lying hollow with the monumental buildings [56] (Fig 6B) may be assumed to be loci for cereal processing, then this setting could hint at a connection between the work performed and the monumental buildings. Maybe the food was processed for actions inside these buildings. The lower-lying hollow with the monumental buildings was accessible via stairs incorporated into the terrace wall, as already pointed out (Fig 6B). Of course, this scenario remains hypothetical at the moment.

### Grinding stones in the monumental circular buildings

We selected the distribution of handstones in monumental building D (Fig 12B) as a case study for layer III, as the other circular buildings are either partly disturbed by post-Neolithic activities (C and H) or incompletely excavated (A and B). The biography of building D is complex, as already discussed above. The completely excavated ring wall with 11 pillars in situ and two central pillars very likely represents the last stage of a long building history. Kurapkat observed traces of a second, older ring wall to the south of the inner wall [56] and a deep sounding immediately to the north of the building revealed a segment of the same wall [95] (Fig 4). Two of the phytolith samples (M11-263 and M11-269) come from that spot. Above we made the case for a partly intentional backfilling of the building (Fig 5, layers 3–8) and the subsequent complete refill through erosion from higher-lying parts of the mound (Fig 5, layer 9–13), followed by five further sloped layers that completely covered the building (Fig 5, layers 14–19). The two uppermost of these layers were disturbed by ploughing. The distribution was analyzed separately for these three zones. Almost three quarters of the handstones lie in the erosion layers (Fig 5, layers 9–19) above the actual filling (Fig 5, layers 1–8). However, the uppermost filling (Fig 5, layers 14–19) contained significantly less handstones than the lower erosion levels (9–13), which produced 51% of all handstones from the building´s area. These layers likely represent dislocated sediments and objects from the surrounding terraces. The distribution is obviously different from that of the rectangular buildings (comp. Fig 12A and 12B with Fig 12C; S2 Table).

Only 30% of the finds lay inside the building´s walls, as they are preserved today (Fig 5, layers 1–8), and only 13% were found in the block of probably intentionally infilled sediments (Fig 5, layers 1–7) above the bedrock on which the building was founded. As mentioned, this particular part of the filling seems to be intentional infill carried out following the last stage of the building´s use-life. All 81 handstones from layers 1–7 (Fig 5) were fragmentary, with the exception of one complete example of type 2, found immediately above the floor (layer 1), which had ochre on its surface (of surface type s5). Three more handstone fragments from here (layer 1) show traces of ochre. Surfaces with ochre and irregular use wear seem thus to be associated with the use of the monumental buildings. We observed both surfaces s1 and s2, as well as irregular surfaces and scar negatives on handstones from the inner filling, in two cases fragments of handstones with surface 2 were reused for the processing of ochre. Processing of ochre and cereals are thus both attestable for layer III, while evidence for ochre is missing so far from layer II. This is interesting, as finds of ochre had already been made in building D. Both central pillars of building D stand in sockets within pedestals cut from the bedrock. In each one of the sockets, sediments mixed with ochre and a fragment of a grinding bowl with ochre traces were discovered. A fragmented plate with ochre stood in front of Pillar 18, the eastern central pillar. No traces of pigments have been observed on the pillars so far, but the insights gained from the study of grinding stones imply that analytical methods should be used to detect such traces in the future. Use of red, white and black pigments is, e.g., attested in contemporary burials from Körtik Tepe [113].

## Discussion

This integrated scientific archaeological approach has for the first time produced a basis for assessing the role of cereals at Göbekli Tepe. The massive presence of grinding equipment and standardization in the production and use of handstones hint at large-scale cereal processing in layer II. This is supported by use-wear traces and the presence of phytoliths in samples from their surfaces.

While charred plant remains are rare, probably due to site formation processes, phytolith analyses verify the significant presence of cereals for all layers at Göbekli Tepe. In building D, however, grinding equipment from the deepest layer, which appears to be connected to the partially intentional refilling of the structure, also shows traces of ochre. The partial contemporaneity of layers III and II, as indicated by radiocarbon data [81] and analyses of building history [56], could mean that cereal processing was conducted mainly in the rectangular buildings. Before the construction of the rectangular buildings, open spaces between or next to the monumental buildings could have served as activity areas for cereal processing. Also, some of the bedrock features on the limestone plateaus surrounding the site [79] are comparable to what has been interpreted as ‘rock-cut mortars’ in the southern Levant [31]. But even if we focus exclusively on the mobile tools used for cereal processing, their apparently high number suggests a high intensity of cereal processing and use throughout the site´s history.

During the experiments we observed that pendula-like bidirectional grinding motions are the most efficient method for grinding and producing fine grained flour. 500 g of einkorn was processed in 40 to 60 minutes, producing around 500 g of flour (ground cereals mixed with stone particles). A single handstone of types 1 or 2 –used bidirectionally–could therefore have produced an average of 4800 g flour within eight working hours. If we assume that one person needs between 500 g and 1000 g of cereals daily as nutrients for survival [46], this amount would be enough to feed five to ten people. However, it is hard to establish the number of grinding stones used contemporaneously at any given phase at the site. Domestic features contemporary to the older phases of the monumental buildings have not yet been clearly identified. Not all layer II buildings were used for domestic activities and, as explained above, it is difficult to determine how many ‘households’ might have been contemporaneous. A more detailed diachronical study comparing different contemporary ‘household’ inventories, as performed by K. Wright [114] for Çatalhöyük, is therefore not feasible at Göbekli Tepe.

However, the overall quantity of 7268 analyzed grinding tools from Göbekli Tepe appears to be too high for simple daily use, given their relatively high productivity. Comparisons to other (partly) contemporary sites are hard to make, as often the total quantity of grinding equipment is not clear from the reports, and while plans show the total area exposed the amount of sediments excavated is not mentioned. Davis described 869 complete or fragmented handstones and 479 grinding slabs (7 whole) for Çayönü [45]. At Jerf el Ahmar, 400 querns were found [30]. Wright´s [114] sample of contextualized material from Çatalhöyük includes 1129 querns / slabs, 26 roughouts and 168 handstones. PPNB assemblages from the Southern Levant do not exceed 500 grinding stones per site, and even Late PPNB assemblages have no more than 1000 grinding stones [115]. There are, however, several factors affecting the distribution and density of grinding stones, like the number of inhabitants of the sites, access to raw materials for their production, the impact of curative technologies on their frequencies, environmental conditions, and culinary preferences. Also, processes of site formation and post-depositional factors impact on the circulation of objects, affecting each comparison between relative and/or absolute frequencies. It is impossible to define the number of grinding stones being used *at the same time*. However, the better preserved and extensively excavated rectangular buildings at Göbekli Tepe have produced an average number of 2 grinding stones/m^3^, which at the actual state of research appears to be very high for the time and region. Building D has an average of 2.45 grinding stones/m^3^. But while there is a high concentration of tools for certain domestic activities like grinding–and also hunting [61]–other categories of material culture are missing from both layers II and III (bone points and awls are very rare, and clay figurines are completely missing) [52]. Only a selection of domestic tasks was performed at the site. As mentioned, there is also no evidence in form of hearths or fireplaces suggesting cooking activities in fixed locations inside or outside the rectangular buildings or in the monumental round structures. When interpreting the massive presence of grinding equipment, it is thus necessary to take the peculiarities of Göbekli Tepe into account, as it differs in various aspects from other Neolithic sites.

Göbekli Tepe has a high concentration of distinctive architecture, often addressed as ‘special buildings’, which do not repeat the characteristic plans of domestic buildings from contemporaneous settlements. Extensively excavated settlement sites like Nevalı Çori [53] or Çayönü [60] have one ‘special building’ per settlement phase, while Göbekli Tepe has several, likely contemporary [56, 64] buildings of this type, which different groups of people likely used. For the buildings excavated so far, we have observed certain regularities governing the decoration of the 69 known pillars–mostly with animal motifs, but also with abstract signs [49, 58, 116]. While in building A snake images prevail, in building B foxes are dominant. In building C boar take over, and in building D the imagery is more diverse with birds, especially vultures, playing a significant role. In building H felines are of importance. We see these differences in figurative expression as evidence for different groups of people ornamenting the buildings with the emblematic animals central to their group identities [49, 58, 116]. Göbekli Tepe has also produced a wide range of stationary and portable art, far outnumbering such finds from other contemporary sites. Many of the animal and human depictions are clearly marked as male, there are almost no female depictions [64, 52], a situation contrary to the materials known from contemporary settlements. Göbekli Tepe´s remote location on a barren mountain ridge is very unusual compared to the setting of contemporaneous Neolithic settlements, which are regularly located next to water sources. No springs are known near Göbekli Tepe. Today, a small seasonal stream, the Mucid Dere, runs at a distance of ca. 3 km to the west; the nearest outlet lakes are located ca. 5 km away at Edene to the northeast and Germuş to the southwest [117]. There is evidence for a system of possibly Neolithic cisterns on the limestone plateaus to the west of the site [117]. The overall capacity of the cisterns found so far is 153 m^3^. This limits the possible number of people permanently present on site, as rainwater would only be constantly available during autumn/winter to refill the cisterns. We see these peculiarities as evidence for a specialized site, with a high concentration of ‘special buildings’. This does not rule out the possibility that a smaller group of people actually lived at Göbekli Tepe permanently. Some of the layer II buildings are domestic in nature, and their existence overlaps with the monumental buildings, even if we still do not know of domestic architecture contemporary to the older phases of the latter.

The construction of monumental architecture at Göbekli Tepe, and other similar sites in its vicinity [118], would have necessitated a workforce of hundreds of people even by conservative estimates [52, 116]. One model to explain cooperation in small-scale communities involves ritualized work feasts. M. Dietler and E. Herbich define work feasts as events in which “commensal hospitality is used to orchestrate voluntary collective labour,” the incentive to work together is provided by the prospect of large amounts of food and drink [119]. Work feasts can mobilize hundreds of people across kinship or friendship networks, they are temporally finite and no obligations remain after their end. The number of people mobilized depends directly on the quantity and quality of food and drink provided. The main archaeological marker for feasting would be evidence of the presence of larger amounts of foodstuffs and tools than needed by the inhabitants of a site for their subsistence. We have presented evidence for Göbekli Tepe that fits that pattern for plant food. To characterize the intense production at Göbekli Tepe, a comparison with Jerf el Ahmar is helpful. Although there are similarities in site structure (rectangular buildings for plant processing next to round buildings), there is one important difference: at Göbekli Tepe no large silos have been identified. Production was not for storage, but for immediate use.

K. Twiss [120] has argued that meat is often the most common food at feasts, and large animals are often of peculiar importance as they provide large amounts of meat and–due to the dangers involved in killing them–also prestige to hunters. For the early Neolithic, she emphasizes the importance of the aurochs, which also plays a big role in PPN imagery and ritual deposits [76]. At Göbekli Tepe, the aurochs takes second place among the hunted species [58] but far more impressive is the amount of gazelle bones [75]. Gazelle is migratory; an easily accessible large-scale supply of gazelle meat was available between midsummer and autumn [75]. The mass of bones recovered hints at mass killings in that short period of the year. While the prestige aspect of hunting large game like aurochs is evident, aurochs also provide lots of fat, essential to people for surviving the winter months, indifferent of how many actually stayed on site [76]. Hunting large numbers of gazelle and Asiatic wild ass, the third important species at Göbekli Tepe, cannot, however, be explained by this “quest for fat” [76]. It is likely that they were hunted to supply a large quantity of meat for seasonal peaks in site use [76], which we identify as work feasts.

Alcoholic beverages are another important aspect of feasts [119, 120], and producing them is an important use of cereals. Tentative evidence for the consumption of alcohol at Göbekli Tepe has been published [51]. Consumption during feasts may be associated with special serving paraphernalia [120]. Göbekli Tepe has produced around 80 sherds of stone drinking vessels [72]. The vessels are thin-walled and made in part of varieties of ‘greenstone’. About half of the fragments were decorated. Stone vessel fragments appear in all strata at Göbekli Tepe; some have marks of repair (holes to fit fragments together) and some sherds were reused as ‘shaft straighteners’. Both likely indicate the high value of the raw material.

Besides food and drink, Twiss identifies a special physical setting, ritual / performance and commemoration as key indicators to prove feasting in the archaeological record [120]. As has been discussed in detail elsewhere, many of these indicators fit the evidence from Göbekli Tepe [51, 72, 73, 116]. At Göbekli Tepe, the non-domestic monumental buildings with their benches hint at a gathering of some sort, while ritual and performance inside or near these buildings are highlighted by evidence in the form of several miniature stone masks, suggesting masquerade [121]. We assume that the stone masks are miniature representations of real organic masks actually worn, as there are further hints towards the importance of masquerade. It was remarked early on that crane depictions at Göbekli Tepe feature human legs–whereas the anatomy of other birds is depicted correctly–and therefore might indeed indicate masked humans [64].

Thus, at Göbekli Tepe, we have evidence of feasting, tentatively including the use of fermented beverages, as an incentive to participate in large-scale construction work. The necessity to provide food and drink for these work feasts would have resulted in the need for large-scale food supplies and their processing at certain times, which would explain the extraordinarily high number of dedicated tools for cereal processing, analyzed for the first time here. K. Schmidt had hypothesized that the amount of food needed for work feasts could have been a contributing factor in the search for more reliable food sources and ultimately domestication [64]. Our study further proves his argument that feasting was an important social practice and provides an explanation for the possibility of large-scale building activities at Göbekli Tepe. However, our findings rather suggest that such feasts were held strategically in seasons favorable to the natural availability of plant food and meat between midsummer and autumn.

Much prior work has focused on Göbekli Tepe´s special character as a cultic center. A new and detailed engagement with those aspects of the site so far not in the center of attention helps to fill in gaps and modify this interpretation. We still believe that the monumental round buildings served ritual purposes and were not just elaborately decorated residential spaces [122], but we have to move towards a more integrative view of domestic and ritual activities at the site [123] using newly available scientific methods and integrating new insights from recent work in the region.

## Supporting information

S1 Table Total amount of analyzed grinding stones (current database). (XLSX)

S2 Table Distribution of grinding stones in the fillings in main excavation area (current database). (XLSX)

S3 Table Distribution of grinding stones in rooms in main excavation area. (XLSX)

S4 Table Description of samples, phytolith amounts, relative abundances of phytoliths, anatomical origin of grass phytoliths and amount of multicell phytoliths obtained from all sediment samples. (XLSX)

S5 Table Morphometric results of the parameter "Largest width" of elongate dendritic phytoliths of selected wheat and barley species from Ball et al. (1999). (XLSX)

S6 Table Morphometric results of the parameter "Largest width" of elongate dendritic phytoliths of selected samples from Göbekli Tepe. (XLSX)

S7 Table Comparison of morphometric results of the parameter "Largest width" of elongate dendritic phytoliths between species measured by Ball et al. (1999) and samples from Göbekli Tepe by the Welch t test. (XLSX)

S8 Table Comparison of morphometric results of the parameter "Largest width" of elongate dendritic phytoliths between Göbekli Tepe samples by the Welch t test. (XLSX)

S9 Table Description of samples, sampling strategy and phytolith amounts obtained from selected grinding stones. (XLSX)

S10 Table List of phytolith morphotypes identified and their frequencies (counts) in soil samples from the L9-69 sequence and a stone vessel, giving the stratigraphic location and sample information. (XLSX)

S11 Table List of phytolith morphotypes identified and their frequencies (counts) in all grinding stone samples, giving sample information and sampling strategy. (XLSX)

S12 Table Numbers and repository information. (XLSX)

S1 Movie Experimental grinding of einkorn to fine flour with pendula-like grinding motions. (MP4)

S2 Movie Experimental grinding of einkorn to coarse flour with circular grinding motions. (MP4)
